# Synergistic Effects of Waste Tire Rubber and Styrene–Butadiene–Styrene on the Viscoelastic Rheology and Fatigue Mechanisms of Asphalt Binders

**DOI:** 10.3390/polym18141750

**Published:** 2026-07-17

**Authors:** Syed Khaliq Shah, Abdullah I. Almansour

**Affiliations:** 1 School of Transportation, Southeast University Road, Jiangning District, Nanjing 211189, China; 233209946@seu.edu.cn; 2 Civil Engineering Department, King Saud University, Riyadh 11451, Saudi Arabia

**Keywords:** waste tire rubber, viscoelastic continuum damage, rheological characterization, fatigue life prediction, storage stability

## Abstract

Conventional rubber-modified asphalt faces critical limitations, including phase separation and compromised low-temperature flexibility, which typically restrict rubber content to 15–25%. This study investigates the viscoelastic rheology and fatigue life prediction of asphalt binders modified with 20% waste tire rubber, utilizing a chemical–thermal desulfurization process and a synergistic styrene–butadiene–styrene (SBS) composite. Five binders were evaluated: base asphalt (BA), SBS-P, WTR, WTD, and WTC. The WTC exhibited the highest softening point at 89 °C and a ductility of 35 cm, successfully overcoming the traditional stiffness–flexibility trade-off. Dynamic shear rheometer (DSR) tests revealed a 440% increase in complex shear modulus (G*) at 58 °C 10.8 kPa vs. 2.0 kPa for BA and a 13.2% reduction in phase angle (61.2°). Multiple stress creep recovery (MSCR) results showed WTC achieved a 60% recovery rate at 0.1 kPa and maintained 25% at 3.2 kPa, with non-recoverable creep compliance (*J_nr_*3.2) well below the 1.0 kPa^−1^ threshold for heavy traffic. Bending beam rheometer (BBR) tests confirmed WTC low-temperature suitability, achieving a creep stiffness of 280 MPa and an *m*-value of 0.58 at −24 °C. Furthermore, viscoelastic continuum damage (VECD) modeling demonstrated that WTC increased yield stress by 80.8% (470 kPa) and extended fatigue life by over two orders of magnitude at low strain (2%) compared to BA. Finally, WTC exhibited excellent storage stability, with a softening point difference (SPD) below 2.2 °C. These findings confirm that the rubber/SBS composite provides a highly durable, storage-stable, and fatigue-resistant binder suitable for long-life asphalt pavements under extreme climatic and heavy-traffic conditions.

## 1. Introduction

The exponential growth in global vehicular traffic has generated a dual infrastructure challenge: accelerating deterioration of flexible pavements and the unprecedented accumulation of end-of-life tires (ELTs) [1]. Annually, over 1.5 billion waste tires are generated worldwide, with a formal recycling rate consistently below 50%, posing severe environmental, public health, and fire hazards [2,3]. Concurrently, asphalt pavements are increasingly subjected to heavier axle loads and more extreme climatic conditions, exacerbating premature failures, such as high-temperature rutting, fatigue cracking, and moisture-induced stripping [4,5]. To mitigate these issues while advancing circular economy principles, the incorporation of waste tire-derived crumb rubber (CR) into asphalt binders has emerged as a globally recognized sustainable solution [6]. Rubber-modified asphalt (RMA) not only valorizes millions of tons of ELTs but also enhances pavement durability, reduces maintenance frequency, and lowers lifecycle energy consumption and carbon emissions [7].

Despite its environmental and economic appeal, conventional RMA faces critical technical barriers that limit its widespread adoption, particularly in long-life asphalt pavement (LLAP) applications [8]. Traditional RMA typically restricts rubber content to 15–25% by weight due to severe workability issues [9], poor storage stability [10], and pronounced phase separation [11] during high-temperature storage and transportation [12]. The vulcanized cross-linked structure of conventional CR impedes homogeneous dispersion in the asphalt matrix, leading to rubber settlement, inconsistent binder performance, and increased mixing temperatures. Furthermore, while conventional CR improves high-temperature stiffness [13,14], it often compromises low-temperature flexibility and fatigue resistance [15], making it inadequate for modern pavements subjected to complex, high-stress traffic loading spectra [16]. These limitations underscore the need for advanced modification strategies that can safely incorporate higher rubber dosages while maintaining rheological balance, storage stability, and structural integrity.

Recent advancements in rubber activation and desulfurization technologies have opened new pathways for high-content rubber utilization. Mechanical–thermal and chemical desulfurization processes effectively break sulfur cross-links in waste tires, producing activated rubber powder with enhanced solubility, reduced viscosity, and superior compatibility with asphalt maltene fractions [17,18,19]. However, desulfurization alone can diminish the elastic recovery of the binder, potentially compromising fatigue performance under repeated loading. To address this, the synergistic incorporation of styrene–butadiene–styrene (SBS) elastomer has been proposed. SBS forms a continuous polymeric network that compensates for elasticity loss, significantly improving creep recovery, temperature susceptibility, and crack propagation resistance [20]. Recent studies have demonstrated that composite binders containing 35% activated rubber powder combined with 2–3% SBS exhibit exceptional high-temperature rutting resistance (*J_nr_*3.2 < 1.0 kPa^−1^), superior storage stability, and fatigue life improvements exceeding 400% compared to conventional RMA [21,22]. The integration of linear amplitude sweep (LAS) testing with viscoelastic continuum damage (VECD) theory has further enabled precise [23,24], mechanistic fatigue life prediction, providing a robust framework for LLAP binder selection [25].

To bridge this knowledge gap, the present study investigates the rheological behavior and fatigue life prediction of high-content waste-tire-derived rubber/SBS composite modified asphalt. Specifically, conventional crumb rubber is subjected to chemical–thermal activation to enhance asphalt compatibility, enabling appropriate rubber dosage without compromising storage stability or workability. The synergistic effect of activated rubber powder and SBS polymer is systematically evaluated through comprehensive dynamic shear rheometer (DSR) testing, multiple stress creep recovery (MSCR) analysis, and accelerated linear amplitude sweep (LAS) experiments. Fatigue damage evolution is modeled using the VECD framework to predict binder fatigue life across varying strain levels.

## 2. Materials and Methods

### 2.1. Base Asphalt

A standard 60/70 penetration grade base asphalt was procured from Attock Refinery Limited (ARL), Morgah, Rawalpindi, Pakistan. This grade was selected due to its extensive use in Pakistani road construction and its balanced properties suitable for the region’s climatic conditions and traffic patterns. The fundamental properties of the base asphalt were evaluated according to ASTM and AASHTO standards, and the results are presented in Table 1. All measured properties confirm to the requirements specified in ASTM D946 and AASHTO M20, confirming its suitability for heavy-traffic flexible pavement applications [26].

### 2.2. Waste Tire Rubber

End-of-life tires (ELTs) were sourced from authorized regional recycling facilities in Pakistan. The tires were mechanically shredded, magnetically separated to remove steel cords, and granulated to a particle size distribution of 40–80 mesh using ambient grinding technology. Prior to modification, the crumb rubber powder (CRP) was thoroughly characterized for essential physical and chemical parameters to ensure material consistency and suitability for asphalt modification [37]. The characterization included determination of ash content, fiber residue, metal content, specific gravity, and proximate composition [38]. The basic properties of the crumb rubber used in this study are presented in Table 2.

### 2.3. Polymer and Additives

Linear styrene–butadiene–styrene (SBS) copolymer SBS-1401, S/B ratio 30/70, export from China and supplied by sinopec yanshan petrochemical was used as the secondary modifier at a dosage of 4% by weight of the total binder to enhance the elastic recovery and high-temperature performance of the composite binder [43]. A naphthenic solubilizer, a liquid rubber oil supplied by National Refinery Limited, Pakistan, was incorporated at 2% of the rubber mass equivalent to 0.4% of the total binder to improve rubber dispersion and compatibility within the asphalt matrix. Fine sulfur powder was employed as a stabilizer at 0.2% of the total binder to promote cross-linking reactions between the activated rubber particles and SBS polymer, thereby mitigating phase separation during high-temperature storage and transportation [44].

### 2.4. Sample Preparation

**Figure 1:**
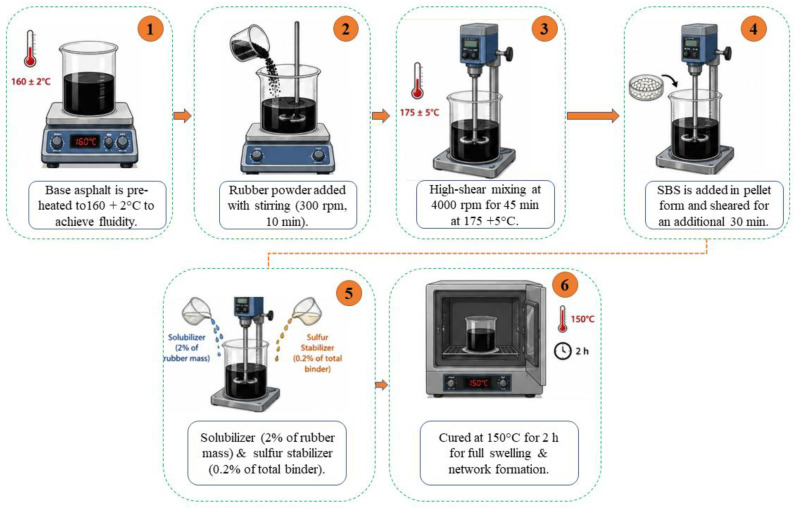
Preparation process for waste tire rubber and SBS composite modified asphalt binders.

Modified binders were prepared using a two-stage high-shear blending protocol. The base asphalt was first pre-heated to 160 ± 2 °C to achieve fluidity. Rubber powder, 20% by weight of the total binder, was gradually incorporated under continuous mechanical stirring at 300 rpm for 10 min, followed by high shear mixing at 4000 rpm for 45 min at 175 ± 5 °C. For the WTC composite blend, SBS pellets, 4% by weight of the total binder, were added and sheared for an additional 30 min. The naphthenic solubilizer, 2% of the rubber mass, and sulfur stabilizer, 0.2% of the total binder, were introduced during the final 10 min of mixing. The preparation process is shown in Figure 1 [45].

All modified binders were subsequently cured at 150 °C for 2 h to ensure complete swelling and network formation. Conventional crumb rubber powder (CRP) was subjected to a controlled thermal–chemical activation process to break vulcanized sulfur cross-links. The powder was treated with a dilute alkaline solution (2 wt% NaOH solution) under controlled heating at 180 °C for 60 min and mechanical agitation, followed by thorough washing and oven-drying. This desulfurization step significantly reduced Mooney viscosity and increased surface polarity, enhancing compatibility with the asphalt maltene fraction while preserving the elastomeric backbone required for fatigue resistance. The detailed sample compositions and nomenclature used in this study are presented in Table 3.

## 3. Experimental Testing Program

All binder-level tests were conducted on unaged and short-term-aged RTFO per AASHTO T240 samples unless otherwise specified. Testing followed recognized ASTM/AASHTO standards to ensure reproducibility and global benchmarking [46].

### 3.1. Conventional and Physical Properties

Penetration (ASTM D5), softening point (ASTM D36), and ductility at 5 °C (ASTM D113) were measured to evaluate basic consistency and low-temperature flexibility. Brookfield rotational viscosity (ASTM D4402) was determined using a Brookfield rotational viscometer equipped with a standard Superpave spindle No. 27 at a rotational speed of 20 RPM, corresponding to a shear rate of approximately 20–25 s^−1^. Measurements were conducted at 135 °C, 150 °C, and 165 °C to assess workability and construction temperature suitability [47].

### 3.2. Storage Stability Test

Phase separation was evaluated per ASTM D5892. Approximately 50 g of each modified binder was poured into aluminum tubes (140 mm height, 25 mm diameter), which were sealed and stored vertically at 163 °C for 48 h. After cooling, the tubes were sectioned into top and bottom halves, and softening points were measured [48,49].

### 3.3. Rheological Tests

The rheological properties of the asphalt binders were evaluated using a DSR and a BBR. DSR testing was conducted using parallel plate geometries of 25 mm diameter with a 1 mm gap for intermediate and high-temperature measurements, and 8 mm diameter with a 2 mm gap for low-temperature evaluations. A temperature sweep test was performed from 58 °C to 88 °C at an angular frequency of 1.0 rad/s and a strain level of 12% to determine the complex shear modulus (G*), phase angle (*δ*), and rutting factor (G*/*sinδ*). Frequency sweep tests were carried out at 60 °C over a frequency range of 0.1–100 rad/s under 10% strain to characterize the viscoelastic behavior and construct master curves. The rutting resistance and elastic recovery performance of the binders were further assessed through the multiple stress creep recovery (MSCR) test in accordance with AASHTO TP70 at 60 °C under stress levels of 0.1 and 3.2 kPa, with ten loading cycles applied at each stress level. The non-recoverable creep compliance (*J_nr_*) and percent recovery (*R*) were determined and used to classify traffic loading capacity based on SHRP/AASHTO specifications. Low-temperature performance was evaluated using the bending beam rheometer (BBR) test according to AASHTO T313 after RTFO aging. Creep stiffness (*S*) and *m*-value were measured at −12 °C, −18 °C, and −24 °C to assess thermal cracking resistance, with performance criteria of S ≤ 300 MPa and m ≥ 0.300 [50,51].

### 3.4. Linear Amplitude Sweep (LAS) Test and VECD Modeling

Fatigue characteristics were evaluated using the linear amplitude sweep (LAS) test per AASHTO TP101. Tests were conducted at 25 °C using an 8 mm parallel plate geometry with a 2 mm gap. A frequency sweep 0.2–30 Hz at a controlled strain of 0.1% was first performed to determine the undamaged complex modulus G*_0_ and the viscoelastic parameter α, where α = 1/*m* and *m* is the slope of the log complex modulus versus log frequency curve [52,53]. Subsequently, a strain-controlled sweep (0.1% to 30% at a constant frequency of 10 Hz) was applied to induce progressive fatigue damage [54].

Damage accumulation was modeled using the viscoelastic continuum damage (VECD) framework. The evolution of the damage parameter, *S*(*t*), was calculated using Equation (1):

(1) $$S\left(t\right)=\sum^{N\;j=1}[\frac{\left[\left(\pi I_{D}{ro}^{2}\right)\right]}{1+a}(C_{j-1}C_{j})\frac{a}{a+1}(t_{j}-t_{j-1})\frac{1}{1+a}]$$

where *I_D_* is the initial integrity parameter (defined as the ratio of the undamaged complex modulus to 1 MPa), γ_0_ is the applied strain amplitude, *t* is the loading time, and *C_j_* is the integrity index at cycle *j*.

The integrity index *C_j_*, which represents the ratio of the damaged to the undamaged dynamic modulus, was determined using Equation (2):

(2) $$C_{j}=\frac{{|G}_{j}^{*}|sin\sigma_{j}}{|G_{0}^{*}|sin\sigma_{0}}$$

where ∣G_j_*∣ and *δ_j_* are the dynamic shear modulus and phase angle at cycle *j* and ∣G*_0_∣ and *δ*_0_ are the initial undamaged values.

The relationship between the integrity index and the accumulated damage parameter was established by fitting the damage characteristic curve using Equation (3):

(3) $$C\left(S\right)=C_{0}-C_{1}S^{C2}$$

where *C*_0_ = 1 represents the undamaged state and *C*_1_ and *C*_2_ are empirical fitting coefficients that characterize the material’s damage resistance.

Finally, the fatigue life prediction (*N_f_*), representing the number of cycles to failure, was calculated based on the fitted parameters using Equation (4):

(4) $$N_{f}=\frac{f{\left(0.5\times {\left(C_{0}÷C_{1}\right)}^{\frac{1}{C_{2}}}\right)}^{1+a-c_{2a}}}{\left(1+a-C_{2a}\right){\left(\pi I_{D}C_{1}C_{2}\right)}^{a}}\gamma^{-2a}$$

where *f* is the loading frequency (10 Hz) and *γ* is the applied strain level. To simulate varying pavement structural responses and evaluate the binder’s fatigue endurance under different traffic conditions, the predicted cycles to failure (*N_f_*) were calculated at applied strain levels of 2%, 3%, 4% and 5.0%.

## 4. Results and Discussion

### 4.1. Conventional Physical Properties

**Figure 2:**
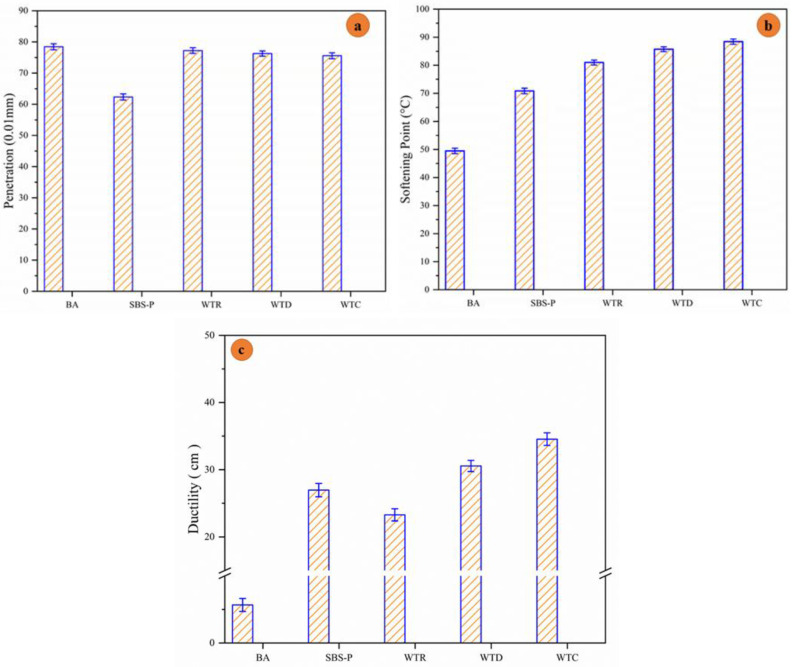
Conventional physical properties of modified binders: (**a**) penetration at 25 °C, (**b**) softening point, and (**c**) ductility.

The conventional physical properties of asphalt binders, including penetration, softening point, and ductility, were evaluated to assess the effectiveness of waste tire rubber and SBS composite modification (Figure 2). The results demonstrate that all modified binders exhibited improved performance compared with the base asphalt (BA), confirming the beneficial influence of rubber activation and SBS incorporation.

The penetration results shown in Figure 2a indicate a reduction in binder consistency after modification, reflecting increased stiffness. The SBS-modified binder SBS-P showed the highest reduction in penetration, while rubber-modified binders exhibited more moderate decreases. Among the rubber-based binders, the activated rubber composite binder WTC achieved the lowest penetration value, suggesting enhanced structural stability while maintaining acceptable workability. The relatively small reduction in penetration despite the high rubber content (20%) highlights the effectiveness of mechano-thermal desulfurization in improving rubber asphalt compatibility and dispersion [55].

Significant improvements were observed in the softening point (Figure 2b), demonstrating enhanced high-temperature stability and rutting resistance. Compared with the base asphalt, all modified binders exhibited substantially higher softening points, with the performance increasing in the order of WTR, WTD, and WTC. The superior performance of WTD compared with WTR confirms that desulfurization improves rubber–asphalt interaction by breaking sulfur cross-links and increasing rubber activity. The WTC binder achieved the highest softening point, indicating a strong synergistic effect between activated rubber powder and SBS. The formation of a three-dimensional polymer network significantly enhanced thermal stability, making the binder highly suitable for heavy-traffic pavements and high-temperature climatic conditions.

Ductility testing further revealed remarkable improvements in low-temperature flexibility (Figure 2c). While SBS-P substantially increased ductility due to its elastomeric characteristics, the activated rubber composite binder exhibited the highest ductility among all samples. The improvement from WTR to WTD demonstrated the positive effect of desulfurization in reducing stress concentration and improving rubber dispersion [56]. The outstanding ductility of WTC indicates that the combination of activated rubber and SBS successfully overcomes the traditional trade-off between high-temperature stiffness and low-temperature flexibility by forming an interpenetrating polymer network that provides both elasticity and structural stability.

### 4.2. Analysis of Brookfield Viscosity

**Figure 3:**
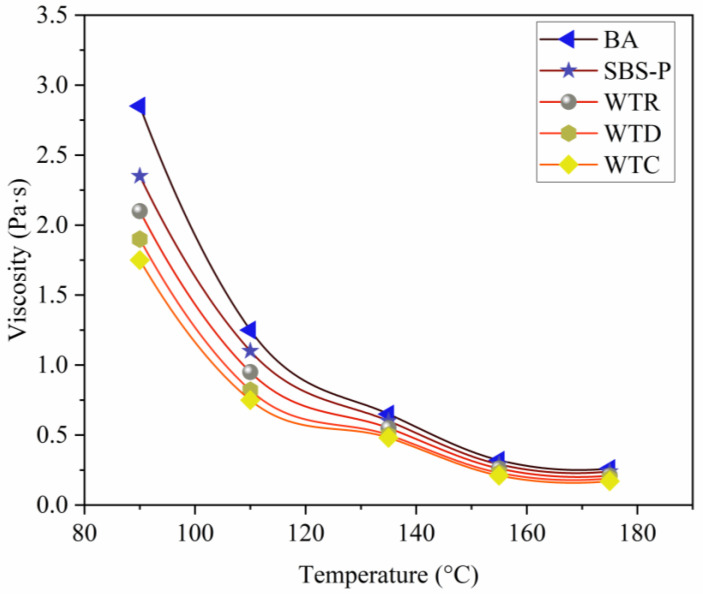
Viscosity–temperature curves of modified binders.

Figure 3 presents the viscosity–temperature relationships of all binders between 80 and 175 °C. All samples exhibited a typical exponential decrease in viscosity with increasing temperature, confirming the thermoplastic behavior of asphalt binders. At 90 °C, viscosity decreased in the order BA > SBS-P > WTR > WTD> WTC, with WTC showing the lowest value. As the temperature increased, viscosity values converged, reaching 0.20–0.30 Pa·s at 175 °C, indicating good workability during mixing and compaction. The lower viscosities of WTD and WTC demonstrate the effectiveness of mechano-thermal desulfurization, which improves rubber dispersion and reduces internal friction within the asphalt matrix. WTC exhibited the best viscosity profile, highlighting the synergistic interaction between activated rubber and SBS. Overall, the results confirm that the activated rubber/SBS composite binder provides improved workability, reduced temperature sensitivity, and excellent constructability despite containing 20% waste tire rubber.

### 4.3. Complex Shear Modulus and Phase Angle

**Figure 4:**
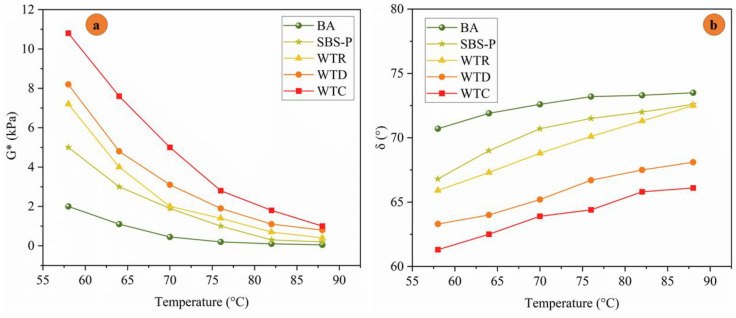
Temperature sweep test results: (**a**) complex shear modulus (G*) versus temperature and (**b**) phase angle (*δ*) versus temperature for modified asphalt binders.

Figure 4 presents the temperature-dependent variations in complex shear modulus (G*) and phase angle (*δ*) of the investigated binders. As the temperature increased from 58 to 88 °C, all binders exhibited a progressive decrease in G* and an increase in *δ*, indicating a reduction in stiffness and a shift toward more viscous behavior. However, the magnitude of these changes differed significantly among the binder formulations [57], highlighting the influence of rubber activation and SBS modification on rheological performance.

The complex shear modulus results revealed a consistent ranking of WTC > WTD > WTR > SBS-P > BA throughout the tested temperature range, as shown in Figure 4a. At 58 °C, the G* value increased from approximately 2.0 kPa for BA to 10.8 kPa for WTC, indicating a substantial enhancement in binder stiffness and resistance to shear deformation. Similarly, WTD and WTR exhibited G* values of approximately 8.2 and 7.2 kPa, respectively, demonstrating that both rubber modification and rubber activation effectively improved the load-bearing capacity of the asphalt binder. The superior performance of WTC suggests that the combined action of activated rubber and SBS generated a more effective viscoelastic network than either modification used independently [58].

The effectiveness of the activation process is evident from the comparison between WTD and WTR. At 58 °C, the G* of WTD was approximately 14% higher than that of WTR, indicating that desulfurization improved rubber–asphalt compatibility and enhanced the reinforcing effect of the rubber phase. The partial cleavage of sulfur cross-links increases the surface activity of rubber particles and facilitates their swelling within the asphalt matrix, resulting in a more homogeneous microstructure and improved stress-transfer efficiency. Similar improvements in complex modulus following rubber activation have been reported in previous studies, confirming the beneficial effect of desulfurization on rheological performance.

The phase angle results further support the G* findings, as shown in Figure 4b. The binders followed the order BA > SBS-P > WTR > WTD > WTC, with WTC consistently exhibiting the lowest *δ* values across the investigated temperature range. At 58 °C, the phase angle decreased from approximately 70.5° for BA to 61.2° for WTC, representing a reduction of 13.2%. Likewise, ref. [59] WTD exhibited a phase angle approximately 4% lower than WTR, confirming that activated rubber contributes to a greater elastic response than untreated rubber. At 88 °C, WTC maintained a phase angle of approximately 66 °C, whereas BA approached 73.5 °C, demonstrating that the composite-modified binder acts synergistically, producing a more stable and resilient rheological structure [60].

The lower *δ* values of WTC indicate that a larger proportion of the applied energy was stored elastically rather than dissipated through viscous flow. This behavior is particularly beneficial for reducing the accumulation of permanent deformation under repeated traffic loading. Compared with SBS-P, WTC exhibited phase angles that were approximately 5–6 °C lower throughout the temperature range, suggesting that the incorporation of activated rubber further enhanced the elastic recovery characteristics provided by SBS alone. This improvement can be attributed to the development of an interpenetrating polymer network in which activated rubber particles and SBS chains interact.

### 4.4. Multiple Stress Creep Recovery (MSCR) Performance

#### 4.4.1. Cumulative Strain Evolution

**Figure 5:**
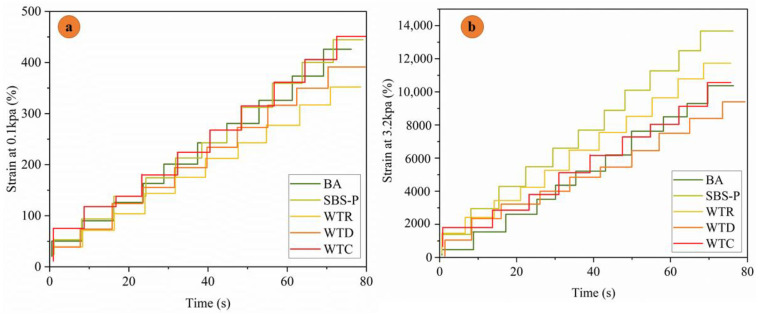
Cumulative strain evolution during the multiple stress creep recovery (MSCR) test at (**a**) 0.1 kPa and (**b**) 3.2 kPa stress levels.

The multiple stress creep recovery (MSCR) test was employed to evaluate the binders’ resistance to permanent deformation and their elastic recovery capacity under simulated traffic loading conditions. The cumulative strain evolution at low stress (0.1 kPa) and high stress (3.2 kPa) is presented in Figure 5a and b, respectively. At the low stress level of 0.1 kPa, all binders remain within the linear viscoelastic region, resulting in relatively modest cumulative strain values below 500% across the ten loading cycles. The strain accumulation curves for BA, SBS-P, WTR, WTD, and WTC are closely clustered, indicating that, under mild loading conditions, the baseline stiffness of all formulations is comparable [61]. Slightly lower strain values are observed for WTD and WTC, suggesting that the incorporation of desulfurized rubber particles enhances the initial elastic resistance even before nonlinear deformation mechanisms are activated.

A pronounced divergence in performance emerges under the high-stress condition of 3.2 kPa, which simulates the severe shear demands imposed by heavy commercial vehicles and slow-moving traffic. At this stress level, the cumulative strain increases substantially, reflecting the transition into the nonlinear viscoelastic regime where permanent deformation dominates. The SBS-P formulation exhibits the highest strain accumulation, reaching approximately 14,000% by the final cycle, indicating that the physical cross-linking network formed by SBS alone is more susceptible to viscous flow under severe stress [62]. In contrast, the rubber-modified binders demonstrate markedly improved resistance to permanent deformation. The WTR shows intermediate strain accumulation, while the WTD achieves the lowest cumulative strain of approximately 9500%. The WTC maintains a similarly low strain trajectory near 10,500%, confirming that the activated rubber network effectively restricts irreversible molecular rearrangement under high shear stress [63].

The stepwise strain profiles also reveal critical insights into the recovery behavior during the rest periods between loading cycles. The vertical strain increments during the 1 s loading phases are consistently smaller for WTD and WTC, while the subsequent recovery segments exhibit steeper relaxation slopes, indicating a higher proportion of elastic strain recovery. This enhanced recovery capacity is directly attributable to the mechano-thermal desulfurization process, which breaks the rigid sulfur cross-links in conventional tire rubber, reduces particle agglomeration, and promotes uniform swelling within the asphalt maltene phase [64]. When combined with the SBS elastomer, the activated rubber particles form an interpenetrating polymer network that efficiently stores and releases strain energy, thereby minimizing residual deformation. The WTR lacks this optimized network structure, resulting in slower recovery and higher permanent strain accumulation under repeated high-stress cycling.

#### 4.4.2. Non-Recoverable Creep Compliance and Percent Recovery

**Figure 6:**
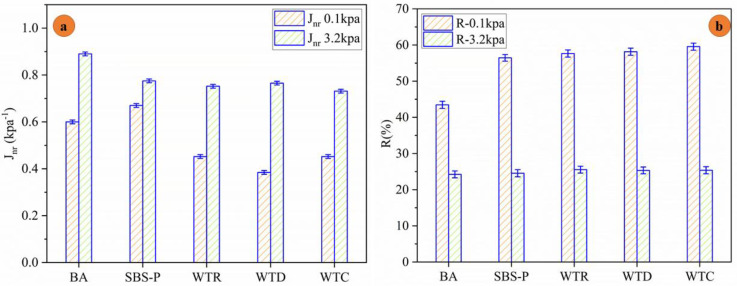
Multiple stress creep recovery (MSCR) test results: (**a**) non-recoverable creep compliance; (**b**) percent recovery.

The non-recoverable creep compliance (*J_nr_*) and percent recovery (*R*) obtained from the MSCR test were used to evaluate the rutting resistance and elastic recovery characteristics of the modified binders. As shown in Figure 6a,b, all binders exhibited stress-dependent behavior, characterized by increasing *J_nr_* and decreasing R values as the stress level increased from 0.1 to 3.2 kPa. This response reflects the progressive development of nonlinear viscoelastic deformation under higher loading conditions.

At 0.1 kPa, WTD exhibited the lowest *J_nr_* value, approximately 0.39 kPa^−1^, indicating the highest resistance to permanent deformation under low-stress conditions. The incorporation of activated rubber significantly reduced the accumulation of unrecoverable strain compared with the unmodified and conventionally modified binders. Meanwhile, WTC exhibited the highest recovery value, approximately 60%, demonstrating superior elastic response and deformation recovery capability. Even at the elevated stress level of 3.2 kPa, WTC maintained a recovery rate of approximately 25%, whereas the recovery of BA decreased substantially, indicating a loss of elastic response under severe loading conditions. The superior performance of WTC can be attributed to the synergistic interaction between activated waste tire rubber and SBS polymer. The desulfurization treatment enhances the compatibility between rubber particles and the asphalt matrix, while SBS contributes a continuous elastomeric network capable of storing and releasing deformation energy. As a result, the composite binder exhibited lower permanent strain accumulation and greater elastic recovery than either rubber modification or SBS modification alone.

A comparison between WTD and WTR further highlights the effectiveness of rubber activation. WTD consistently exhibited lower *J_nr_* values and higher recovery percentages than WTR at both stress levels, indicating that the activation process improved the efficiency of stress transfer within the binder and strengthened the internal viscoelastic network [65]. The improved performance is attributed to the increased surface activity and enhanced swelling behavior of the desulfurized rubber particles, which promote stronger interactions with the asphalt components.

The stress sensitivity of the binders can also be assessed through the variation between *J_nr_*0.1 and *J_nr_*3.2. WTC and WTD exhibited relatively small increases in *J_nr_* with increasing stress compared with BA, indicating greater resistance to stress-induced structural degradation and improved stability under heavy traffic loading. According to the AASHTO M332 specification, binders with *J_nr_*3.2 values below 1.0 kPa^−1^ are suitable for very heavy to extremely heavy traffic conditions. All modified binders satisfied this requirement, whereas WTC exhibited the most favorable combination of low *J_nr_* and high recovery.

### 4.5. Low-Temperature Rheological Performance

**Figure 7:**
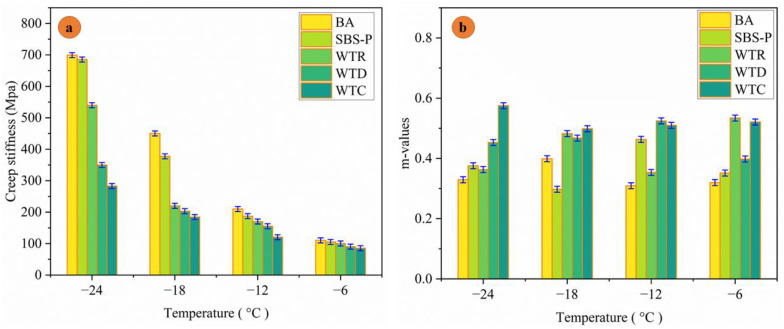
Low-temperature rheological properties from BBR testing: (**a**) creep stiffness (*S*) and (**b**) *m*-value of base and modified asphalt binders at temperatures ranging from −24 °C to −6 °C.

The results for creep stiffness (*S*) and the *m*-value are presented in Figure 7a and b, respectively. These parameters are critical indicators of pavement performance in cold climates, where binders must remain flexible to withstand thermal contraction stresses without fracturing.

Figure 7a illustrates the creep stiffness of the binders at temperatures ranging from −24 °C to −6 °C. As expected, all binders exhibit a characteristic increase in stiffness as the temperature decreases, reflecting the transition towards a more rigid, glass-like state. However, the magnitude of this stiffening varies significantly among the formulations. The BA demonstrates the highest stiffness across all temperature levels, reaching approximately 700 MPa at −24 °C, which far exceeds the Superpave specification limit of 300 MPa, indicating a high susceptibility to brittle fracture in cold conditions. In contrast, the modified binders, particularly WTC and WTD, exhibit substantially lower stiffness values. At −24 °C, WTC achieves a stiffness of approximately 280 MPa, and WTD reaches roughly 350 MPa, both representing a significant reduction in rigidity compared to BA. This improvement confirms that the activation of waste tire rubber and the incorporation of SBS effectively disrupt the rigid molecular structure of the asphalt, maintaining binder flexibility even at extreme low temperatures [60]. The WTR shows an intermediate stiffness of 540 MPa, suggesting that, while crumb rubber provides some stiffening, it is the activated rubber combined with SBS that truly enhances low-temperature compliance.

Figure 7b presents the *m*-value, which characterizes the binder’s capacity for stress relaxation. A higher *m*-value indicates that the binder can dissipate internal stresses more effectively, thereby reducing the likelihood of thermal cracking. The results show that WTC consistently maintains the highest *m*-values across the tested temperature range, reaching approximately 0.58 at −24 °C and maintaining values above 0.50 at −12 °C. In comparison, the base asphalt (BA) exhibits the lowest relaxation capacity, with *m*-values hovering around 0.32 at −24 °C and dropping to 0.31 at −6 °C. The superior relaxation behavior of WTC can be attributed to the synergistic network formed by the desulfurized rubber and SBS elastomers [66]. This network allows the binder to deform and recover elastically under thermal stress, rather than storing energy that leads to crack propagation.

An interesting deviation in the stress relaxation capacity is observed in the *m*-value trends at specific intermediate low temperatures. For the conventional rubber binder WTR, a noticeable drop in the *m*-value occurs at −12 °C. This anomaly is attributed to the rigid, unbroken sulfur cross-links within the conventional crumb rubber particles. As the temperature approaches −12 °C, these unmodified rubber particles undergo a localized stiffening or glass transition, transforming from compliant inclusions into rigid constraints that restrict the viscoelastic relaxation of the surrounding asphalt matrix. Conversely, for the desulfurized binder WTD, an unexpected drop in the *m*-value is observed at −6 °C. This behavior can be explained by the extensive swelling of the highly activated rubber particles at this relatively warm low temperature. The desulfurized rubber absorbs a significant portion of the maltene light fractions from the asphalt matrix to facilitate swelling [67]. This localized depletion of light components increases the viscosity and stiffness of the continuous asphalt phase, temporarily reducing its stress relaxation rate (*m*-value) at −6 °C before the binder exhibits superior relaxation capacity at much lower temperatures (−18 °C and −24 °C), where the entire composite system behaves more uniformly.

According to Superpave performance grading criteria, a binder is considered suitable for low-temperature applications if *S* ≤ 300 and *m* ≥ 0.300. The WTC formulation satisfies both criteria at −24 °C, *S* ≈ 280 MPa, 0.58, qualifying it for use in severe cold climates potentially meeting a PG grade such as PG 70-28 or similar depending on high-temp results. Conversely, the base asphalt fails the stiffness criterion at −18 °C and −24 °C, limiting its application to warmer regions.

### 4.6. Fatigue Performance Analysis Using Linear Amplitude Sweep and VECD Theory

**Figure 8:**
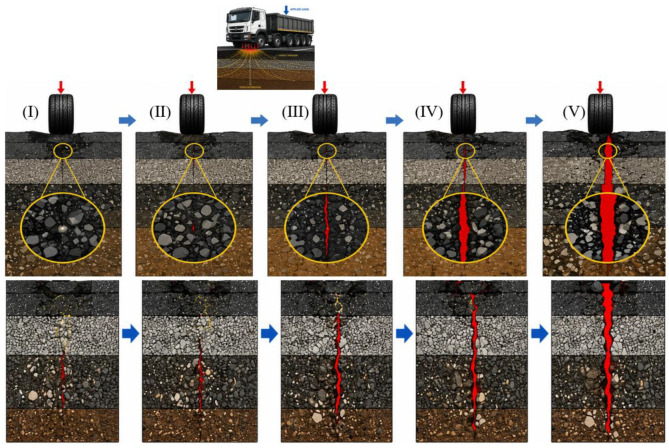
Pavement-level fatigue damage progression: stress distribution beneath wheel load and typical tensile strain response showing stable response, micro-crack initiation, crack propagation, and fatigue failure stages.

To contextualize the binder-level fatigue evaluation, Figure 8 illustrates the multi-scale progression of fatigue damage in asphalt pavements subjected to repeated heavy traffic loading. The figure depicts the characteristic five-stage damage evolution from initial stress concentration and micro-crack initiation at the bottom of the asphalt layer Stage I–II, through upward crack propagation and coalescence Stage III–IV, to eventual full-depth structural failure Stage V. This bottom-up cracking mechanism, driven by tensile strain accumulation, represents the primary distress mode that the LAS testing and VECD modeling aim to quantify [68]. By simulating these progressive damage states at the binder level, the VECD framework provides a mechanistic prediction of fatigue life that directly correlates with the macroscopic pavement performance.

#### 4.6.1. Shear Stress–Strain Response and Yield Characteristics

**Figure 9:**
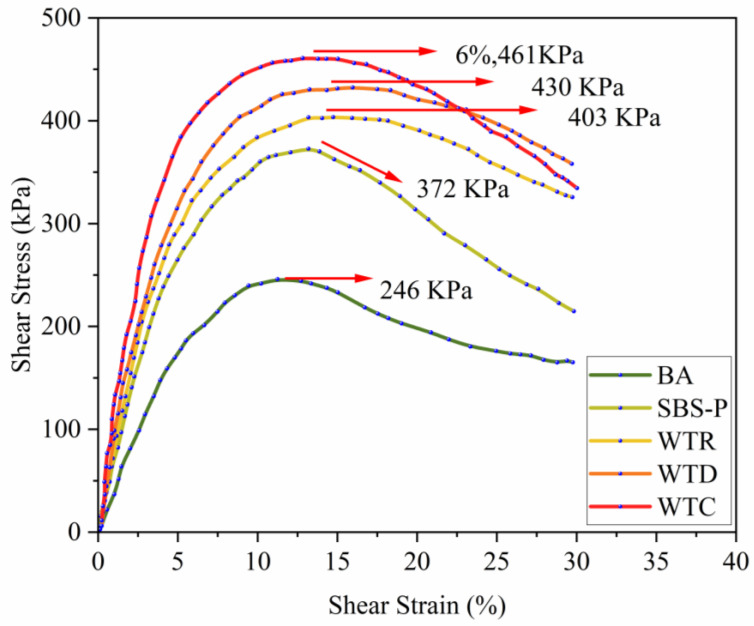
Shear stress–strain relationships from LAS testing, highlighting the peak yield stress and corresponding failure strain for the investigated binders.

The shear stress–strain relationships for all binder types are presented in Figure 9, revealing critical insights into their yield behavior and damage tolerance.

All binders exhibit a characteristic nonlinear response, with shear stress increasing rapidly during the initial loading phase before reaching a peak value yield stress, *τmax*, followed by a gradual decline as damage accumulates and micro-cracks propagate. The peak stress values demonstrate a clear performance hierarchy: WTC 461 kPa > WTD 430 kPa > WTR 403 kPa > SBS-P 372 kPa > BA 246 kPa. This represents an 87.4% increase in yield stress for WTC compared to BA, indicating substantially enhanced resistance to shear deformation under repeated loading [69]. The superior yield strength of the composite binder is attributed to the robust interpenetrating polymer network formed by the synergistic interaction between activated rubber particles and SBS, which effectively restricts molecular flow and delays the onset of macroscopic yielding.

The post-peak behavior reveals critical differences in damage propagation mechanisms. BA exhibits a steep stress decline after reaching *τmax*, indicating a rapid loss of load-bearing capacity and brittle-like failure with accelerated crack propagation. In contrast, WTC demonstrates a significantly more gradual stress reduction, reflecting enhanced ductility and the ability to sustain high stress levels even after initial damage initiation. This ductile post-peak behavior is crucial for pavement applications, as it allows the binder to accommodate thermal stresses and traffic loading without catastrophic failure [70]. The activated rubber particles in WTC act as compliant inclusions that blunt crack tips and promote energy dissipation through viscoelastic deformation, while the SBS network bridges developing micro-cracks. Consequently, the WTC composite fundamentally shifts the failure mode from rapid brittle fracture to a gradual, damage-tolerant cracking mechanism, thereby extending the fatigue life of the pavement.

#### 4.6.2. Damage Characteristic Curves Based on VECD Theory

**Figure 10:**
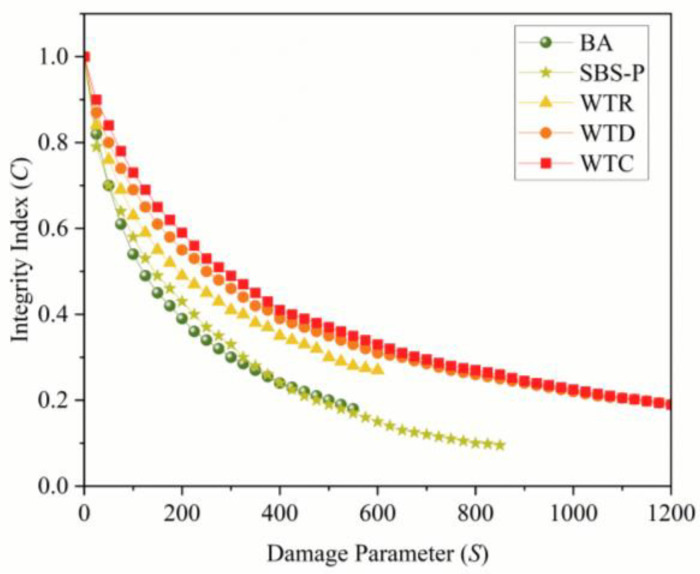
Damage characteristic curves (integrity index C vs. damage parameter S) for modified binders based on VECD theory from LAS testing.

The VECD framework provides a mechanistic basis for characterizing fatigue damage evolution in asphalt binders. The integrity index (C) versus damage parameter (S) curves, presented in Figure 10, show the progressive degradation of binder stiffness as a function of accumulated damage [71].

The integrity index C, defined as the ratio of damaged to undamaged complex modulus, decreases monotonically with increasing damage parameter S for all binder types. However, the rate of degradation varies significantly among formulations. At any given damage level, the integrity hierarchy follows: WTC > WTD > WTR > SBS-P > BA. For instance, at S = 400, WTC maintains C ≈ 0.45, whereas BA has degraded to C ≈ 0.25, indicating that WTC retains 80% more structural integrity under equivalent damage conditions.

**Figure 11:**
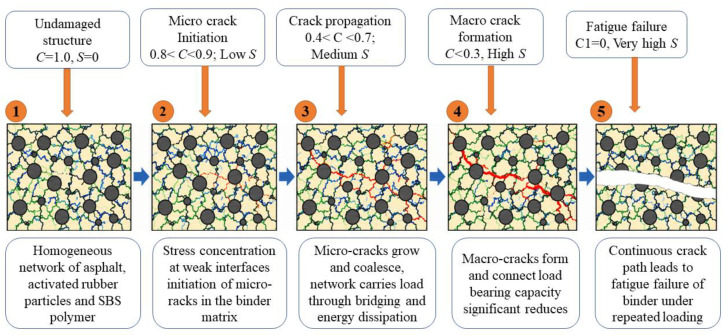
VECD-based fatigue damage evolution and crack propagation mechanism in modified asphalt binder.

The progressive damage evolution in asphalt binders follows a characteristic five-stage mechanism, as illustrated in Figure 11. Starting from an undamaged homogeneous network Stage 1, C ≈ 1.0, S ≈ 0, the binder undergoes micro-crack initiation at weak interfaces Stage 2, 0.8 ≤ C < 0.9, followed by crack propagation and coalescence Stage 3, 0.4 ≤ C < 0.7, macro-crack formation Stage 4, C < 0.3, and finally complete fatigue failure Stage 5, C → 0. The VECD framework mathematically captures this progression through the integrity index C(S), where slower degradation of C with increasing S indicates superior damage tolerance [72].

The superior damage tolerance of WTC is particularly evident in the initial damage phase S < 200, where the C–S curve exhibits a relatively gentle slope compared to other binders. This indicates that the activated rubber/SBS composite network effectively resists early-stage micro-crack formation and propagation. The mechano-thermal desulfurization process enhances rubber–asphalt compatibility, allowing activated rubber particles to swell uniformly and form strong interfacial bonds with the asphalt matrix. When combined with SBS elastomers, these particles create an interpenetrating polymer network that distributes stress concentrations and delays the coalescence of micro-cracks into macro-cracks.

The extracted VECD fitting parameters (*C*_1_, *C*_2_), critical damage values *S_f_*, defined at a failure threshold of *Cf* ≈ 0.1, and predicted fatigue life at 2.5% strain.

The parameter *C*_1_ represents the initial rate of damage accumulation, while *C*_2_ dictates the acceleration of damage as micro-cracks coalesce. The damage growth kinetics, represented by the rate of integrity loss (*dC*/*dS*), reveal fundamental differences in failure mechanisms among the binders. As detailed in Table 4, the WTC binder exhibited the lowest *C*_1_ value (0.0042) and the highest *C*_2_ exponent (0.68) among all formulations. A lower *C*_1_ indicates a highly resistant initial microstructure with a remarkably suppressed initial damage evolution rate (*dC*/*dS*), while a higher *C*_2_ mathematically confirms that the binder can sustain a significantly larger damage parameter before catastrophic failure. Consequently, WTC achieved the highest critical damage value (*Sf* = 1850), representing an increase in damage tolerance compared to the BA (*Sf* = 680). This expanded failure envelope confirms that the WTC composite fundamentally alters the damage progression, allowing it to absorb cyclic energy without rapid structural collapse [73].

#### 4.6.3. Fatigue Life Prediction Model Development and Validation

**Figure 12:**
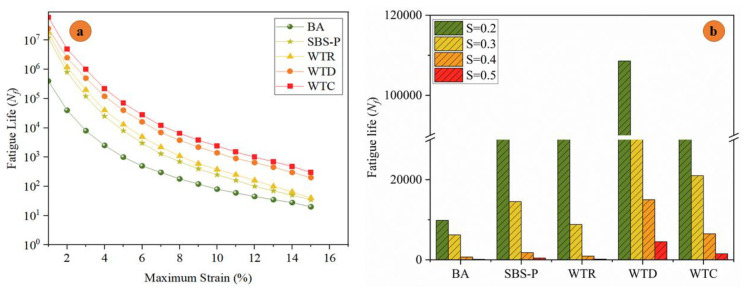
VECD-based fatigue life prediction: (**a**) fatigue life (*N_f_*) as a function of maximum applied strain and (**b**) fatigue life at discrete damage thresholds (SS = 0.2, 0.3, 0.4, and 0.5).

The fatigue life (*N_f_*) of the modified binders was predicted using the VECD framework, with results evaluated as a function of maximum applied strain and discrete damage thresholds. Figure 12a illustrates the strain-dependent fatigue behavior on a logarithmic scale, revealing a characteristic power-law decay where predicted fatigue life decreases exponentially with increasing strain amplitude. Across the entire strain spectrum 2–15%, all modified formulations consistently outperform the base asphalt (BA), with the composite binder WTC demonstrating the highest fatigue resistance. At low strain levels (2%), WTC achieves a predicted life exceeding 10^6^ cycles, which is approximately two orders of magnitude greater than BA. As strain increases to 10%, the performance divergence becomes more pronounced [74], WTC maintains a fatigue life above 10^3^ cycles, whereas BA drops below 100 cycles. This sustained superiority across low-to-high strain conditions confirms that the synergistic combination of activated waste tire rubber and SBS effectively delays micro-crack initiation and preserves structural integrity under repeated traffic loading.

Figure 12b further elucidates the damage tolerance of each binder at discrete damage/strain thresholds, S = 0.2 to 0.5, simulating progressive deterioration under varying severity levels. At low damage states (S = 0.2), the WTD exhibits the highest fatigue life, which is primarily attributed to the enhanced initial elasticity and uniform particle dispersion achieved through the activation process. However, as the damage parameter increases to S = 0.4 and 0.5, representing advanced micro-crack accumulation and higher stress concentrations, WTC demonstrates superior retention of fatigue life compared to WTD and other formulations. This shift in performance hierarchy underscores the critical mechanistic role of SBS within the composite matrix. While activated rubber optimizes low-strain resilience and initial stiffness, the SBS elastomer establishes a continuous polymeric network that bridges developing micro-cracks, dissipates strain energy, and retards damage propagation under severe loading conditions. Consequently, WTC offers a more balanced and robust fatigue profile, maintaining elevated cycle counts even at advanced damage states where conventional and single-modifier binders experience rapid degradation.

To understand why WTC accumulates damage more slowly and exhibits an extended failure envelope, it is essential to link the macroscopic VECD parameters to the microscopic damage growth kinetics. The slower damage accumulation (lowest *C*_1_ of 0.0042) and delayed catastrophic failure (highest S*_f_* of 1850) of WTC are rooted in the synergistic interpenetrating polymer network (IPN) formed during the chemical–thermal desulfurization and SBS compounding. Under cyclic fatigue loading, this dual-phase network retards damage growth kinetics through two primary mechanisms [75].

First, crack blunting and energy dissipation: The uniformly dispersed, activated rubber particles act as compliant inclusions that blunt propagating crack tips. They promote energy dissipation through viscoelastic hysteresis, preventing localized stress concentrations from reaching the critical threshold required for micro-crack initiation. This directly manifests as the gentle initial slope of the *C−S* curve and the exceptionally low *C*_1_ parameters.

Second, micro-crack bridging and elastic recovery: As damage (*S*) accumulates, the continuous SBS polymeric network acts as a physical bridge across developing micro-cracks. The SBS network provides rapid elastic recovery during the rest periods of the loading cycle, effectively pulling micro-crack faces together and retarding their coalescence into macro-cracks.

Consequently, the WTC binder fundamentally shifts the failure mode from the rapid, brittle fracture observed in BA (characterized by steep *C−S* trajectories and a low *S_f_* of 680) to a gradual, damage-tolerant cracking mechanism. This explains the mathematically observed lower *C*_1_ and higher *S_f_* as the binder can sustain extensive internal micro-damage while maintaining macroscopic load-bearing capacity and structural integrity.

**Figure 13:**
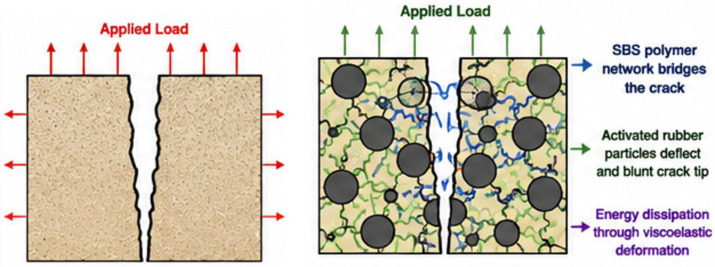
Crack-bridging and energy dissipation mechanism in WTC composite binder.

The mechanistic basis for WTC superior damage tolerance is illustrated in Figure 13, which contrasts the crack propagation behavior of base asphalt with the WTC composite binder. In conventional BA, cracks propagate freely through the matrix with high stress concentration and minimal energy dissipation, resulting in brittle failure and short fatigue life. In contrast, the WTC composite exhibits multiple crack-arrest mechanisms: (1) the SBS polymer network physically bridges developing cracks, maintaining load transfer across crack faces; (2) activated rubber particles deflect and blunt crack tips, reducing stress intensity factors; and (3) the viscoelastic deformation of the composite network dissipates strain energy that would otherwise drive crack propagation.

Table 5 presents the fitted fatigue prediction equations for all investigated binders along with statistical goodness-of-fit parameters. The regression analysis demonstrates excellent correlation between predicted and modeled fatigue life, with coefficient of determination R^2^ values exceeding 0.99 for all formulations. This confirms the robustness of the power-law relationship in characterizing strain-dependent fatigue behavior across the investigated strain range 2–15%.

The fatigue exponent (*k*_2_) provides critical insight into strain sensitivity. Base asphalt exhibits the highest exponent (3.82), indicating extreme sensitivity to strain variations; a small increase in strain results in dramatic reductions in fatigue life. In contrast, WTC demonstrates the lowest exponent (2.94), reflecting superior damage tolerance and reduced strain sensitivity. This 23% reduction in the fatigue exponent signifies that WTC maintains functional performance over a wider range of pavement structural conditions and traffic loading severities. The coefficient *k*_1_ represents the theoretical fatigue life at 1% strain and serves as a comparative indicator of intrinsic fatigue resistance. WTC achieves a *k*_1_ value of 2.8 × 10^8^, which is approximately 180 times greater than that of base asphalt 1.5 × 10^6^. This substantial improvement confirms that the activated rubber/SBS composite fundamentally enhances the binder’s capacity to withstand repeated loading cycles.

**Figure 14:**
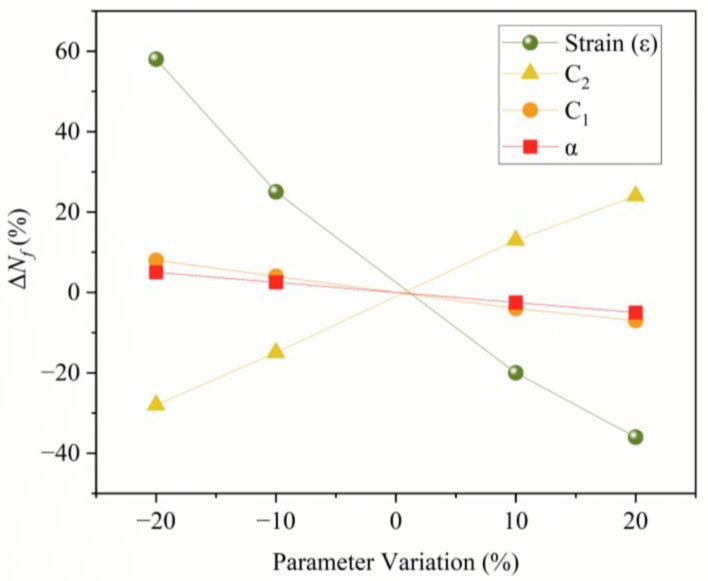
Sensitivity analysis of the VECD-based fatigue life prediction model.

To evaluate the robustness of the fatigue prediction model, a comprehensive sensitivity analysis was conducted to quantify the influence of input parameter variability on predicted fatigue life. Figure 14 illustrates the sensitivity of the model to parameter variations for the WTC binder at a reference strain level of 5%. The results reveal that fatigue life is highly sensitive to variations in applied strain (ε), exhibiting the steepest slope in the sensitivity plot. A ±10% change in strain amplitude produces an approximate ±20–25% change in predicted Nf, while a ±20% variation results in a 36–58% change. This high sensitivity underscores the critical importance of accurate strain determination in pavement structural design. The VECD damage exponent (C_2_) exhibits moderate sensitivity, with ±10% variations resulting in 13–15% changes in fatigue life. Conversely, the initial damage coefficient (C_1_) and the viscoelastic parameter (α) show minimal sensitivity (±10% variation results in <±5% change in *N_f_*), confirming that the model is highly robust to minor uncertainties in early-stage damage characterization and linear viscoelastic properties.

#### 4.6.4. Fatigue Damage Evolution Mechanisms

**Figure 15:**
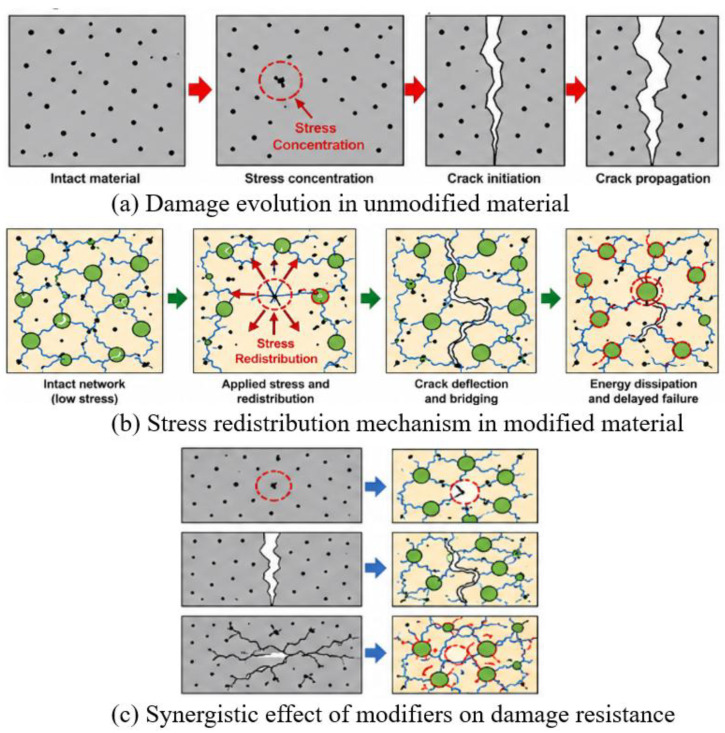
Schematic illustration of fatigue damage evolution mechanisms.

Figure 15 illustrates these mechanisms, contrasting the brittle failure of base asphalt with the damage-tolerant behavior of the composite system. In unmodified base asphalt, as shown in Figure 15a, the homogeneous matrix lacks reinforcing networks, leading to rapid stress concentration at micro-defects and brittle crack propagation with minimal resistance. Conversely, the WTC composite introduces a heterogeneous microstructure where activated rubber particles (green circles) and SBS polymer networks (blue lines) form an interpenetrating polymer network (Figure 15b). This network fundamentally alters damage evolution through four synergistic mechanisms.

First, crack initiation is delayed through stress redistribution. When applied stress develops, the intact network redistributes localized concentrations outward (red arrows in Figure 15b), preventing them from reaching critical thresholds for crack nucleation. This explains the lowest C_1_ value of 0.0042 for WTC in the VECD analysis. Second, crack propagation is resisted through deflection and bridging. Unlike the straight crack path in BA, propagating cracks in WTC must navigate around rubber particles, creating tortuous paths that increase fracture surface area. Simultaneously, the SBS network bridges crack faces, maintaining load transfer and counteracting crack opening. This mechanism explains why WTC maintains *C* > 0.2 even at *S* > 1000, whereas BA fails rapidly at *S* ≈ 600.

Third, energy dissipation is enhanced through viscoelastic hysteresis. The activated rubber particles undergo substantial deformation under cyclic loading, with the desulfurization process increasing molecular mobility and enabling extensive energy dissipation through internal friction. The SBS network’s physical cross-links continuously break and reform during deformation, dissipating additional energy through molecular slippage. This reduces the strain energy available for crack propagation, contributing to WTC’s higher critical damage parameter (*S_f_* = 1850). Fourth, elastic energy storage and recovery are superior due to the dual-phase network. The SBS elastomer functions as molecular springs that store strain energy during loading and release it during unloading, while activated rubber particles provide additional recoverable deformation capacity. This enables WTC to recover substantial strain during rest periods between traffic loads, allowing micro-cracks to close and reducing effective strain ranges in subsequent cycles.

The synergistic effect is evident in Figure 15c; while base asphalt exhibits high stress concentration and rapid failure and rubber modification alone WTD provides moderate improvement through crack deflection, the composite WTC achieves extensive stress redistribution, crack bridging, and energy dissipation. This multi-mechanism approach fundamentally transforms fatigue damage evolution from rapid brittle fracture to gradual, damage-tolerant cracking, mathematically manifesting as the highest *C*_2_ exponent (0.68) and fatigue life exceeding 10^6^ cycles at 2% strain.

#### 4.6.5. Fracture Parameter Evolution

**Figure 16:**
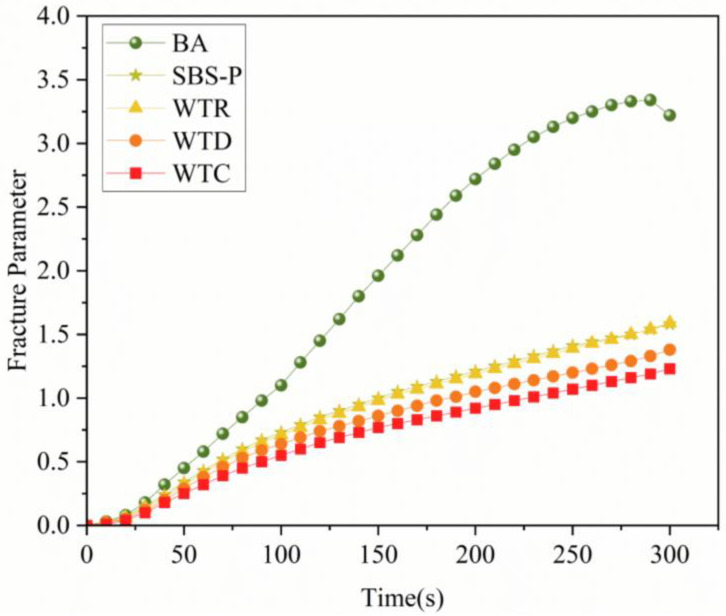
Fracture parameter development over time for the investigated asphalt binders under sustained fatigue loading.

The time-dependent evolution of the fracture parameter, presented in Figure 16, provides critical insights into the crack propagation dynamics and fracture resistance of the modified binders under sustained fatigue loading. The BA exhibits a rapid and nearly linear increase in the fracture parameter over time, reaching approximately 3.3 at 300 s, which signifies accelerated crack growth and a transition toward brittle fracture behavior. In contrast, all modified formulations demonstrate substantially suppressed fracture parameter development, with the WTC showing the slowest progression, stabilizing near 1.25 at 300 s. The WTD and WTR also exhibit marked improvements over BA, with WTD outperforming WTR due to enhanced rubber–asphalt compatibility and reduced internal stress concentration points. This hierarchical resistance to fracture propagation directly correlates with the previously observed fatigue life predictions and damage tolerance characteristics. The superior performance of WTC can be attributed to the synergistic interaction between activated waste tire rubber and SBS polymer, which collectively form a ductile, energy-dissipating matrix that effectively blunts propagating crack tips, redistributes localized stress concentrations, and delays the transition from micro-crack initiation to macroscopic fracture.

The desulfurization process facilitates uniform rubber dispersion and stronger interfacial bonding, while the SBS elastomer provides continuous elastic recovery that counteracts crack opening under cyclic loading. Consequently, the gradual and controlled increase in the fracture parameter for WTC indicates a fundamental shift from brittle to ductile fracture mechanics, significantly extending the binder fatigue endurance. From a practical pavement engineering perspective, this suppressed crack propagation behavior translates directly to reduced bottom-up cracking, longer maintenance intervals, and enhanced structural longevity, particularly under the repeated heavy axle loads and thermal cycling conditions prevalent in Pakistani highway networks. The fracture parameter evolution thus serves as a mechanistic validation of the composite binder’s suitability for long-life asphalt pavement applications, confirming that high-content activated rubber/SBS modification fundamentally alters the failure mode from rapid brittle fracture to gradual, damage-tolerant cracking.

### 4.7. Storage Stability and Phase Separation

**Figure 17:**
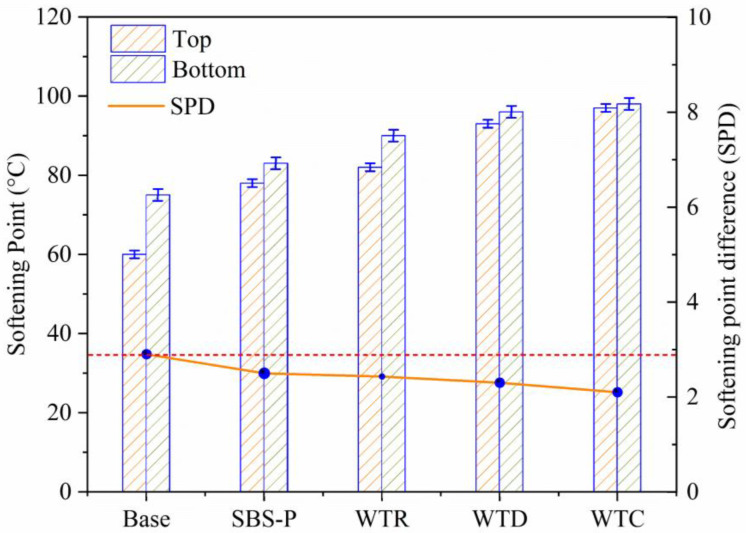
Softening point difference (SPD) and storage stability results for BA, SBS-P, WTR, WTD, and WTC binders.

The thermal storage stability of the modified binders was evaluated to determine their suitability for practical field application, where phase separation during storage and transportation can severely compromise performance. Figure 17 presents the softening points of the top and bottom sections of the storage tubes, along with the calculated softening point difference (SPD).

The softening points of all modified binders are significantly higher than those of the base asphalt, consistent with the conventional property results discussed previously. The modified binders exhibit softening points ranging from approximately 75 °C SBS-P to nearly 100 °C WTC. Generally, the softening points of the bottom sections are slightly higher than the top sections for all samples, which is a typical observation in high-temperature storage tests due to the potential settling of heavier particles or modifiers.

The SPD is the critical parameter for assessing stability, with the red dashed line in Figure 17 representing the standard acceptance criterion, typically 2.5 °C or 3.0 °C depending on the specification. The base asphalt exhibits an SPD exceeding this limit (3.5 °C), indicating poor thermal stability under the test conditions. However, all modified binders demonstrate SPD values well within the acceptable range, confirming that the modification process effectively mitigates phase separation.

The SPD follows a distinct decreasing trend: Base > SBS-P ≈ WTR > WTD > WTC.

The superior stability of WTC is attributed to the synergistic interaction between the activated rubber powder and the SBS polymer. The desulfurization process reduces the density difference between the rubber particles and the bitumen, preventing rapid sedimentation. Furthermore, the SBS elastomer forms a continuous, filamentous network structure within the binder. This network acts as a physical barrier that holds the rubber particles in suspension, effectively preventing phase separation even at high temperatures.

The WTC binder exhibited the lowest softening point difference among all formulations, achieving a reduction of approximately 40–60% compared with conventional rubber-modified asphalt. This improvement confirms that chemical activation significantly enhances compatibility between rubber particles and asphalt components, while the SBS network provides additional stabilization against particle settlement. Consequently, the composite binder maintained excellent storage stability despite containing a high rubber dosage of 20%.

## 5. Conclusions

This study evaluated the rheological properties, storage stability, and fatigue performance of asphalt binders modified with conventional WTR, WTD, and activated waste tire rubber/SBS composite binder WTC. Based on the experimental findings, the following conclusions can be drawn:

1. The activation of waste tire rubber and its combination with SBS significantly improved the conventional properties of asphalt binders. The WTC binder exhibited the highest softening point and ductility while maintaining acceptable penetration, indicating enhanced thermal stability and flexibility.
2. Rheological characterization demonstrated substantial improvements in high-temperature performance. At 58 °C, the complex shear modulus (G*) of WTC reached approximately 10.8 kPa compared with about 2.0 kPa for the base asphalt, while the phase angle decreased from 70.5° to 61.2°, confirming the development of a stronger and more elastic binder network.
3. MSCR testing revealed superior rutting resistance and elastic recovery for the activated rubber formulations. WTD achieved the lowest *J_nr_* ≈ 0.39 kPa^−1^, whereas WTC exhibited the highest recovery rate (60% at 0.1 kPa) and maintained favorable performance under the severe loading condition of 3.2 kPa. All modified binders satisfied the AASHTO requirements for heavy traffic applications.
4. Low-temperature performance was significantly enhanced by rubber activation and SBS incorporation. The WTC binder achieved a creep stiffness of approximately 280 MPa and an *m*-value of approximately 0.58 at −24 °C, satisfying Superpave criteria and demonstrating excellent resistance to thermal cracking.
5. LAS testing showed that WTC exhibited the highest yield stress (470 kPa), representing an increase of approximately 81% compared with the base asphalt. VECD damage analysis indicated that WTC retained substantially greater structural integrity during fatigue loading and exhibited slower damage accumulation than the other binders.
6. VECD-based fatigue life prediction demonstrated that the WTC binder achieved fatigue lives exceeding 10^6^ loading cycles at low strain levels and maintained superior fatigue resistance across all investigated strain amplitudes. The synergistic interaction between activated rubber particles and SBS effectively delayed crack initiation and propagation.
7. Storage stability assessment confirmed that the activated rubber/SBS composite binder exhibited the lowest softening point difference and remained well below the specification threshold, indicating excellent resistance to phase separation despite the high rubber content.
8. From an engineering perspective, the activated waste tire rubber/SBS composite binder demonstrated a unique combination of rutting resistance, thermal cracking resistance, storage stability, and fatigue durability. Therefore, the proposed WTC formulation represents a promising sustainable binder technology for long-life asphalt pavements subjected to heavy traffic loading and extreme climatic conditions while simultaneously promoting the high-value utilization of waste tire resources.

## Figures and Tables

**Table 1:** Physical and rheological properties of base asphalt.

Property	Test Method	Unit	Requirement	Test Result
Penetration at 25 °C	ASTM D5 [27]	0.1 mm	60–70	62
Softening Point	ASTM D36 [28]	°C	46–54	49
Ductility at 25 °C	ASTM D113 [29]	cm	≥100	102
Brookfield Viscosity (135 °C)	ASTM D4402 [30]	Pa·s	≥0.16	0.58
Dynamic Viscosity (60 °C)	ASTM D2171 [31]	Pa·s	≥160	246
Specific Gravity	ASTM D70 [32]	-	-	1.029
Flash Point	ASTM D92 [33]	°C	≥230	242
Fire Point	ASTM D92	°C	≥240	247
Solubility in Trichloroethylene	ASTM D2042 [34]	%	≥99.5	99.6
Wax Content	ASTM D721 [35]	%	≤2.2	1.5
Performance Grade (PG)	AASHTO M320 [36]	-	-	PG 64-22

**Table 2:** Physical and chemical properties of crumb rubber powder.

Property	Unit	Test Method	Value
Particle size	mesh	Sieve analysis	40–80
Specific gravity	-	ASTM D297 [39]	1.18
Bulk density	kg/m^3^	ASTM D1895 [40]	480
Metal content	%	ASTM D5603 [41]	0.01
Fiber content	%	ASTM D5603	0.14
Ash content	%	ASTM D5603	4.5
Natural rubber	%	ASTM D5603	33.5
Synthetic rubber	%	ASTM D5603	8.3
Carbon black	%	ASTM D5603	27
Acetone extractables	%	ASTM D5603	2.1
Carbon (C)	%	EDX	53.6
Oxygen (O)	%	EDX	14.4
Silicon (Si)	%	EDX	14.5
Sulfur (S)	%	EDX	3.1
Calcium (Ca)	%	EDX	7.4
Zinc (Zn)	%	EDX	2.2
Aluminum (Al)	%	EDX	1.6
Mooney viscosity (ML 1 + 4 @ 100 °C)	-	ASTM D1646 [42]	39

Note: The sum of the listed ASTM D5603 proximate analysis components is 75.55%. The remaining 24.45% consists of moisture, volatile matter, and trace unquantified additives not fully isolated by these specific test categories.

**Table 3:** Sample nomenclature.

Sample Code	Description
BA	Base Asphalt (Control)
SBS-P	SBS Polymer Reference
WTR	Waste Tire Rubber (20% conventional)
WTD	Waste Tire Desulfurized (20% activated)
WTC	Waste Tire Composite (20% activated + SBS)

**Table 4:** VECD damage characteristic fitting parameters.

Binder	*C*_1_ (Initial Damage Rate)	*C*_2_ (Damage Acceleration Exponent)	Critical Damage Parameter, *S_f_*	Predicted *N_f_* at 2.5% Strain (Cycles)
BA	0.0452	0.41	680	5.4 × 10^3^
SBS-P	0.0385	0.45	760	1.1 × 10^4^
WTR	0.0215	0.52	1150	3.2 × 10^4^
WTD	0.0128	0.58	1420	7.5 × 10^4^
WTC	0.0042	0.68	1850	1.4 × 10^4^

**Table 5:** Fatigue life prediction equations and statistical parameters for modified binders.

Binder	Fatigue Prediction Equation	R^2^	Fatigue Exponent (*k*_2_)	Coefficient (*k*_1_)
BA	*N_f_* = 1.5 × 10^6. ε−0.382^	0.994	3.82	1.5 × 10^6^
SBS-P	*N_f_* = 4.2 × 10^6. ε−0.351^	0.996	3.51	4.2 × 10^6^
WTR	*N_f_* = 1.8 × 10^7. ε−0.328^	0.993	3.28	1.8 × 10^7^
WTD	*N_f_* = 5.6 × 10^7. ε−0.315^	0.995	3.15	5.6 × 10^7^
WTC	*N_f_* 2.8 × 10^8. ε−0.294^	0.998	2.94	2.8 × 10^8^

## Data Availability

The original contributions presented in this study are included in the article. Further inquiries can be directed to the corresponding author.
